# Is the timing of radiological intervention and treatment day associated with economic outcomes in DRG-financed health care systems: a case study

**DOI:** 10.1186/s12913-017-2055-0

**Published:** 2017-02-28

**Authors:** Christoph Napierala, Stefan Boes

**Affiliations:** 1 Department of Health Sciences and Health Policy and Center for Health, Policy and Economics, University of Lucerne, Frohburgstrasse 3, Luzern, 6002 Switzerland; 2 Siemens Healthcare AG, Zürich, 8048 Switzerland

**Keywords:** Radiology, DRG, Weekday, Weekends, Compliance of length of stay, LOS

## Abstract

**Background:**

In 2012, Switzerland has introduced a diagnosis related group (DRG) system for hospital financing to increase the efficiency and transparency of hospital services and to reduce costs. However, little is known about the efficiency of specific processes within hospitals. The objective of this study is to describe the relationship between timing of radiological interventions, in particular scan and treatment day, and the length of stay (LOS) compliance in a hospital.

**Methods:**

This is a cross-sectional observational study based on administrative records of all DRG cases in a Swiss university hospital in 2013, enriched by data from the radiology information system and accounting details. The data are analysed using descriptive statistics and regression methods.

**Results:**

Radiology and related treatment on a weekend is associated with a higher LOS compliance of approximately 22.12*%* (*p*<0.01) compared to scans and treatments on weekdays, controlling for gender, age and insurance of the patient, as well as detailed medical and radiology-related factors. The higher LOS compliance is driven by emergency cases, which supports the hypothesis that for those cases on weekends more efficient scan and treatment processes are in place.

**Conclusion:**

The study provides evidence on how days of radiological intervention are related to LOS compliance in a Swiss hospital under DRG and attempts to explain how this is linked to standardised operating procedures. Our results have implications regarding potential cost savings in hospital care through alignment of care processes, infrastructure planning and guidance of patient flows.

**Electronic supplementary material:**

The online version of this article (doi:10.1186/s12913-017-2055-0) contains supplementary material, which is available to authorized users.

## Background

### Current discussion

Current research in hospital financing aims at both cost controlling and quality improvement [1, 2]. Clinical processes, including radiology, may be structured more efficiently to increase hospital performance and quality of services provision. Research on the efficiency of radiology contingent on the timing of clinical interventions is rare [3]. However, preliminary research [4] indicates that the day of diagnostic testing might be related to the compliance of length of stay (LOS) and through that to the economic outcomes of a hospital in a diagnosis related group (DRG) system. There is selective evidence on how the timing of admission and intervention can influence economic or clinical results in a hospital environment, e.g., for patients with upper gastrointestinal bleeding [5], mortality from myocardial infarction [6], mortality in general [7–9], and diagnostic productivity of hospitals in DRG systems [8].

This paper aims at describing the relationship between radiological intervention days and LOS compliance in a Swiss university hospital. The underlying hypothesis is that radiological interventions (i.e., scans and related treatments) on weekends as opposed to weekdays have a positive influence on hospital performance in both resource use and quality of care by affecting compliance of LOS. We use LOS compliance as an indicator of hospital performance, process management and quality of care. It is calculated as a benchmark LOS relative to the actual LOS, and we assume that the higher this ratio, the better the hospital performance compared to a benchmark. LOS compliance is considered an adequate dimension of investigation especially in the context of radiology [10]. We also aim at avoiding the shortcomings of the often used LOS dimension which in our view does not adequately deal with process efficiency and quality improvements as there is no reference or output dimension attached.

A higher LOS compliance is indicative of two beneficial mechanisms. First, the hospital uses fewer resources because patients leave the institution earlier than the benchmark. Second, it is reasonable to assume that the quality of care is better. Hospitals strive to be more efficient and avoid patient readmission by providing high quality care along the clinical pathway. DRGs prevent recurrent cases by fixing a time frame (18 days in Switzerland) in which the returning patient with the same main diagnosis will be attributed to the same case. Such a mechanism sanctions the hospital by generating higher costs for the same case. The intrinsic interest of the hospital should therefore be to limit such patients. A potential explanation for the variation in LOS compliance is the timing of treatments along the clinical pathway, i.e., on what day the patient is admitted, diagnosed, treated and discharged [11, 12]. In this study, we describe how radiological interventions on specific days are linked to LOS compliance, based on administrative data of DRG cases in a Swiss university hospital.

### Relevant health system facts

The Swiss health system is rated among the best but also most costly health systems in the world [13]. Despite its good performance, there is potential for improvement, and a major reform in recent history was the implementation of SwissDRG, the Swiss version of a DRG system, on January 1, 2012. With the introduction of a nationwide DRG system, policy makers have received a powerful toolbox to measure, control and anticipate potential inefficiencies in the hospital sector. Moreover, it has become easier to compare hospitals and hospital services through the creation of an implicit benchmarking system.

Before 2012, there was a mixed system in place with some hospitals using a case-mix financing structure (the “All Patient DRG” or APDRG system, which was implemented in 1998), and others using a fee-per-day reimbursement^1^; see [14] for an evaluation of the impact of APDRG on LOS development, and [15, 16] for a general evaluation of the impact of case-mix systems.

A common characteristic of DRGs is the classification of diagnostic or treatment areas into groups that have common traits both clinically and from a cost perspective. Patient characteristics like gender and age also influence the groups. DRG systems apply a certain case weight (CW) to such groups, which indicates the average cost of all cases of this type. Thus, the larger the single CW is, the more severe the case in general. The case weight is multiplied by the respective base rate to obtain the revenue for a particular case in the hospital. The attribution of groups is usually done through a grouper software maintained and documented by a case-mix office (SwissDRG AG in Switzerland).

Besides the average cost per group, the average LOS (ALOS) plays a crucial role in managing the system. ALOS allows for the planning and controlling of health services provision by geographic areas or by single hospitals [17]. It can also be used as a proxy for medical progress [14]. However, one has to be careful in the assessment of the single value pairs per DRG group as they are defined dynamically and their individual weight or reference value changes over time (i.e., the composition is being re-calculated every year). It thus seems to be more promising to use a system of key performance indicators, e.g., the case-mix index, the case weight per case reference values or the LOS compliance to enable a benchmark-oriented comparison. In this study, we will focus on the LOS compliance as indicator for hospital performance.

While the main aim of the DRG implementation by the Swiss Federal Office of Public Health (FOPH) has been to increase transparency of hospital services, contain costs and improve access to health services, there were also other measures relevant to the overall policy adaptation. The following points summarise the main aspects of the policy change:

- Introduction of prospective payment system (DRG) (common tariff structure and hospital related base rates, inclusion of investment costs)
- Dual-fixed financing of remuneration (min. 45% by health insurers, max. 55% by canton)
- New criteria for hospital planning (cantonal hospital lists and service mandates including intercantonal coordination)
- Free choice of hospital for patients
- Transparency measures (e.g. publication of hospital key data and quality indicators)

While the full impact of the reform are still under evaluation, it was expected to lead to a higher degree of competition between hospitals [18–22].

To this end, hospitals should aim at improvements in their process management to strengthen their relative market position and to benefit from the fixed revenues per case. One would also expect a shift towards a more quality-oriented services provision and more in-depth cost assessments, which could be achieved for example by standardised clinical pathways [23, 24]. In the medium to long run, such developments will likely influence the hospital landscape regarding the number of hospitals and the quality of health services provision due to concentration processes and delegation of services to the best performing institutions ([20, 21]). While there is some international evidence on the positive impacts of DRG on the LOS and quality of care [18, 25, 26], evidence for Switzerland is still very limited. A notable exception is [27] who find evidence for a shift from inpatient to outpatient care after the introduction of the DRG system.

### Research interest from a hospital management perspective

Our research interest stems from a dialogue with hospital management and other stakeholders who suspect a relationship between economic outcomes (e.g., profitability, LOS compliance) and clinical interventions (in our case radiological intervention days). We will focus on LOS compliance as we deem it more relevant for the evaluation of clinical pathways. LOS compliance is directly related to profitability, though, via quality improvements and higher efficiency [22].

**Fig. 1:**
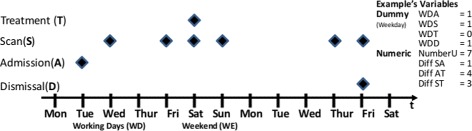
Exemplary patient pathway. Showing a simplified patient pathway including all days of relevance to the analysis and their abbreviations (*Working Day = WD + Type*)

Figure 1 provides an example of the clinical pathway we refer to. We distinguish between admission day, scan day(s), intervention day(s) and discharge day. We hypothesize that the timing of scan and treatment day has an influence on the LOS compliance. We approach the hypothesis from an empirical perspective and seek to increase the evidence base regarding the relationship between medical intervention days and economic and procedural outcomes in modern led hospitals [28]. We also seek to evaluate previous evidence stating that medical technologies (such as imaging diagnostics) do not represent a cost driver for a health system overall but rather a leverage to improve patient pathways [29]. This will also provide evidence-based insights on how hospital processes are currently run and could be improved in the future.

## Methods

### Data sources and definition of variables

The data for this study stem from three different data sources. The reference dataset contains administrative records of 14’026 DRG cases in the hospital under investigation in 2013. This corresponds to the number of cases delivered to the case-mix office with each of them containing at least the four main traits of the patient pathway in Fig. 1, and especially the radiologic scan day. Our case example can be compared to the other university hospitals in Switzerland by its similar case weight and average length of stay. The second data source is the radiological information system (RIS) containing 38’198 interventions, which are collapsed by case and merged to the DRG case file. The third data source is the hospital’s accounting system and DRG cost calculations, which are also added to the data.

In the data cleaning step, we did not restrict our selection to any Major Diagnostic Categories (MDCs) as we deem it relevant to keep all clinical cases. Knowing that some of them cannot be actively influenced through good practice in clinical processes, which is the focus of our research, this implies that our estimates provide a lower bound for the potential process improvements. We excluded cases with a case weight beyond 10 (226 cases) and below 0.1999 (58 cases). We assume that these cases are too special for our analysis and that their distinctly different patient pathways are not comparable to the ones in which an interdependence between the diagnostic interventions, treatments and clinical outcomes can be suspected. In addition, to reduce reporting error, we eliminated cases with implausible differences between admission day and first scan day (18 cases).

Our variable of interest, the LOS compliance, links the effective length of stay per case to a benchmark value. Thus, it indicates whether the institution is providing the DRG services better than the benchmark, in our case this is the ALOS catalogue value of the case-mix office. The LOS compliance for case *i* is formally defined as

1 $$  {LOS compliance}=ALOS_{(DRG,CH)/LOS_{i} }  $$

where the Swiss DRG catalogue ALOS, or reference ALOS value per DRG, is divided by the effective length of stay per case in the hospital. Our basic assumption is that the higher the ratio is, the better it is from a hospital performance and a clinical outcomes perspective. First, it is a sign of good process management within the hospital as cases are treated in less time than the national benchmark. Second, we deem it relevant with respect to an overall health care production function. One can assume that the higher the ratio is, the more economical overall patients are treated. Thus, all else being equal, hospitals with a higher LOS compliance are more efficient and spend less scarce resources on the same health care service on cases.

Even though we suspect cases with a LOS compliance higher than 10 (62 cases) as not relevant for our analysis, or potentially misclassified, we kept them in the data set. After all data cleaning, we remain with a final sample of 11’371 cases for the analysis.

Collapsing the RIS data into DRG cases required to logically combine the intervention category. To characterise the cases appropriately we have extracted the first intervention as the leading one, which is thus also the one deciding upon the efficiency degree of the entire case. Combining it with the total number of interventions and combined with what we call the Scan Category (*SC*), we deem this approach appropriate to sufficiently reflect the variety of interventions. *SC* is defined as a dichotomous variable divided into single (*S*) and complex (*C*) interventions. The reference category is (*S*) and contains single use of a modality without further investigation, e.g., one conventional radiography. All the (*C*) cases reflect sequences of investigations involving one or more modalities, e.g., one use of computer tomography (*CT*) and one or more subsequent uses of further installations like conventional radiography, i.e., x-rays (*XR*), magnet resonance imaging (*MRI*), or sonography (*SON*). In general, we assume that examinations for *MRI*, *CT*, *SON* are more complex than *XR*, as the last modality does not require the same skill level in reading images, for example.

Table 1 summarises the distribution of the number of cases and LOS compliance by first modality and day of scan. We observe the expected drop in interventions on weekends, although *XR* and *CT* services in particular include weekend emergency room interventions as well, and thus the total number of cases for these modalities is significantly higher than for *MRI* and *SON* services. The pattern reflects that elective interventions mainly take place on weekdays (Mon-Fri). Regarding LOS compliance by modality and day of the week, we observe that the LOS compliance tends to be higher for weekend than weekday scans, and that there is substantial heterogeneity between modalities. *MRI*s tend to have the highest LOS compliance, followed by *CT*, *SON* and *XR*. This could be explained by the more technically challenging investigations following *MRI*, which allow for improved clinical patient pathways. The result underpins our differentiation between single and complex radiology interventions to control for the use of more complex modalities to gain process efficiency.

**Table 1:** Number of scans and mean of LOS compliance per modality and weekday

		LOS Compliance							
		Mon	Tue	Wed	Thu	Fri	Sat	Sun	All
MRI	n	271	298	274	206	189	84	24	1346
	Mean S.E.	1.90±0.101	1.81±0.090	1.89±0.110	2.02±0.164	1.68±0.129	2.33±0.320	1.97±0.315	
SON	n	78	77	90	83	104	26	28	486
	Mean S.E.	1.89±0.335	1.36±0.141	1.21±0.118	1.35±0.168	1.40±0.169	1.74±0.341	1.37±0.219	
UCT	n	498	523	471	499	451	279	290	3011
	Mean S.E.	1.68±0.104	1.58±0.080	1.75±0.085	1.65±0.077	1.67±0.096	1.88±0.144	1.84±0.116	
XR	n	1382	1297	1216	1060	845	401	327	6528
	Mean S.E.	1.46±0.047	1.42±0.037	1.40±0.041	1.45±0.046	1.36±0.049	1.46±0.084	1.60±0.119	
	All	2229	2195	2051	1848	1589	790	669	11371

In addition to the scan day, we are interested in the interaction between scan and treatment day and its potential association with the LOS compliance. Table 2 shows the number of cases and the LOS compliance for weekday versus weekend scans and treatments. Out of the 11’371 cases, about 83.33*%* have both first scan and treatment on a weekday, and about 6.39*%* of the cases have scan and treatment on a weekend. The LOS compliance is substantially higher on average (95*%*−*C* *I*=2.195±0.1188) if both scan and treatment are on a weekend compared to both on a weekday (95*%*−*C* *I*=1.549±0.034), and even more so if first scan and treatment day are not coordinated both on a weekend or weekday.

**Fig. 2:**
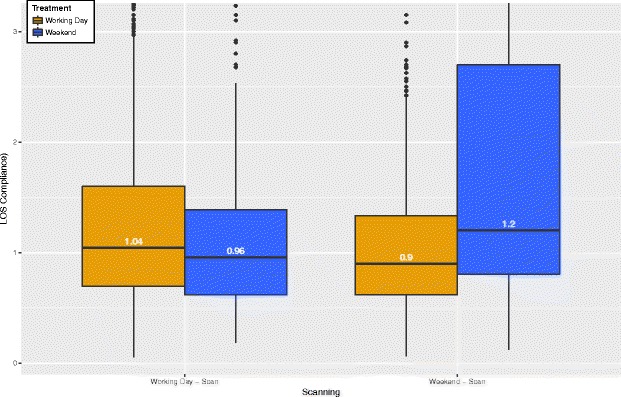
LOS compliance(log) per scanday vs. treatment day. Highlights the difference per day of week and indicates significant differences as the mean and median fluctuate

**Fig. 3:**
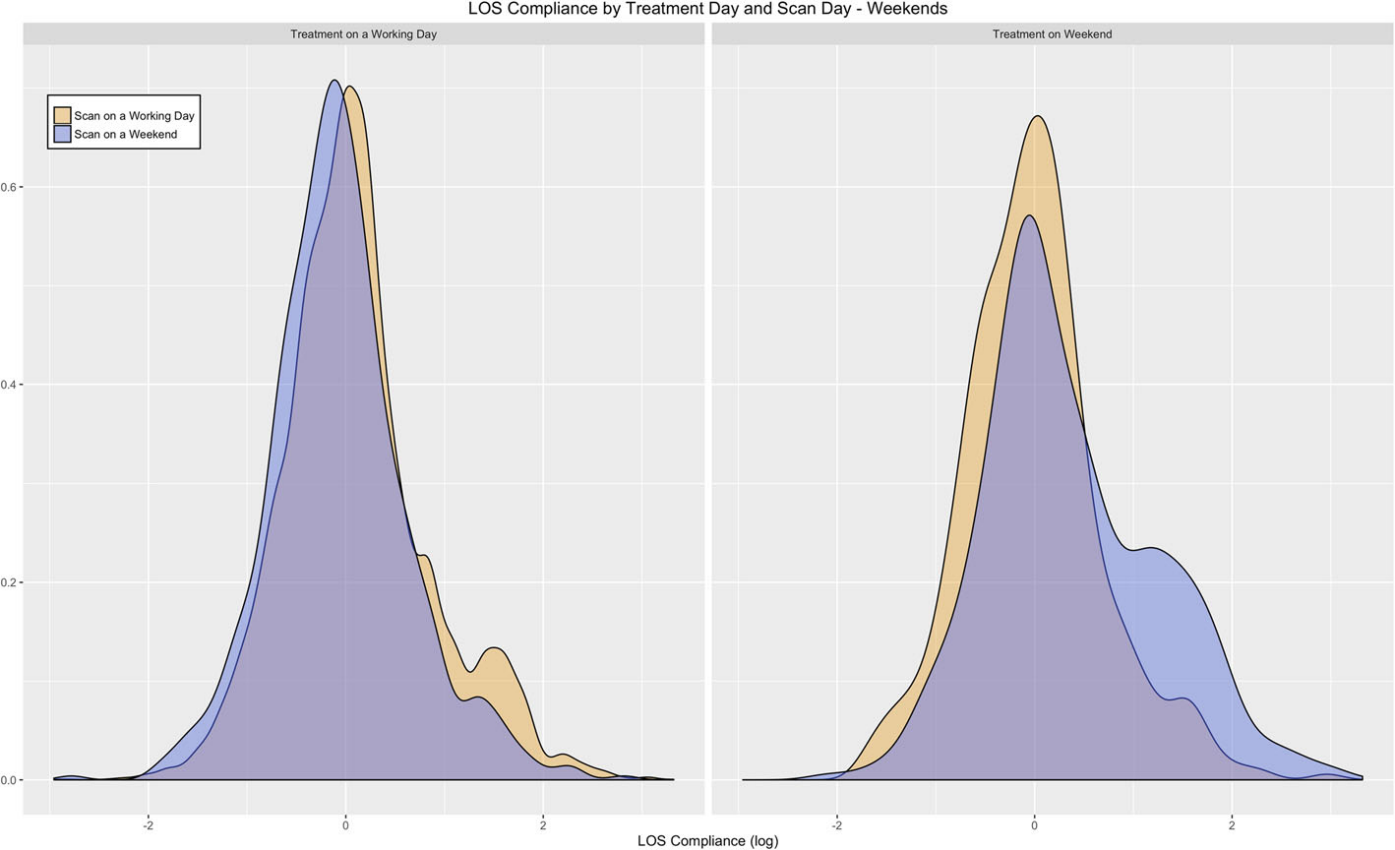
LOS compliance(log) per modality and type of week day. Shows that distributions on both types of day are very similar per modality

Since the distribution of LOS compliance is right-skewed, we use the natural logarithm of LOS compliance to further investigate the differences between weekend scan and treatment. Figures 2 and 3 display the distributions of the log LOS compliance by scan and treatment day (weekday versus weekend), overall and by modality. The distributions indicate overall a very similar shape, but a slight right-shift of the log LOS compliance on weekends, confirming the result for the larger mean LOS compliance on weekends shown in Table 2.

**Table 2:** LOS compliance comparison by working day and weekend of scan vs. treatment

		Working Day		Weekend Treatment	
		LOSC	log(LOSC)	LOSC	log(LOSC)
Working Day Scan	n	9476		436	
	%	83.33		3.84	
	Mean S.E	1.55±0.017	−0.11±0.008	1.31±0.069	−0.01±0.033
Weekend Scan	n	732		727	
	%	6.44		6.39	
	Mean S.E	1.23±0.047	−0.07±0.025	2.19±0.096	0.36±0.032

LOSC = LOS Compliance

Considering that LOS compliance is also determined by other factors potentially related to the scan and treatment day, we include several control variables in our empirical analysis. First, to reflect the clinical pathway illustrated in Fig. 1 as close as possible, we include indicators for weekend admission and discharge. In addition to the total number of scans, we include the time (in days) elapsed between admission day and scan day, between treatment day and first scan day, and between discharge day and first scan day. To further describe the particularities of the case, we add indicators for emergency cases, whether cases refer to internal medicine (as opposed to surgery), and indicators for the complex multi-modality C cases, and the type of modality in the first scan (*XR*, *CT*, *MRI* with reference *SON*) in the empirical model below. Finally, we add the patient’s age at admission (in years) and indicators for female, private insurance coverage, and death during the hospital stay to the set of controls. Table 3 below summarises the mean values of these variables, again by weekend versus weekday scan and treatment.

**Table 3:** Covariate comparison split by working day and weekend of scan vs. treatment

		Working day treatment	Weekend treatment
Working Day Scan	Age	63.31	64.29
	Female	0.48	0.47
	Private	0.27	0.28
	Complex	0.82	0.83
	Mortality	0.06	0.04
	Number of Exams	2.65	2.49
	Emergency	0.85	0.49
	Diff. Treatment Scan Day	−1.61	−0.58
	Diff. Scan Admission Day	3.48	2.73
	Diff. Dismissal Scan Day	6.51	7.71
	CT	0.25	0.25
	MRI	0.12	0.13
	Sonography	0.05	0.04
	X-Ray	0.57	0.59
Weekend Scan	Age	66.17	65.20
	Female	0.45	0.46
	Private	0.27	0.30
	Complex	0.86	0.90
	Mortality	0.04	0.03
	Number of Exams	2.93	3.13
	Emergency	0.89	0.78
	Diff. Dismissal Scan Day	8.30	7.62
	Diff. Scan Admission Day	1.52	3.36
	Diff. Treatment Scan Day	−1.09	0.24
	CT	0.45	0.33
	MRI	0.10	0.05
	Sonography	0.04	0.04
	X-Ray	0.41	0.58

Note: The above numbers do represent mean values for continuous variables or the share of the respective variable referred to the total of cases

### Empirical model

To test the hypothesis whether weekend scans and weekend treatments have an influence on the LOS compliance, we specify the following log-linear regression model

2 $$ \begin{array}{rr} log(LOS compliance_{i}) = &\\ \beta_{0} + \beta_{1}{WEscan_{i}} + \beta_{2}{WEtreat_{i}} &\\ + \beta_{3}{WEscan_{i}*WEtreat_{i}} + \chi_{i} '\gamma+ \epsilon_{i}&\\  \end{array}  $$

where *l* *o* *g* *L* *O* *S* *c* *o* *m* *p* *l* *i* *a* *n* *c* *e*_*i*_ is the log of the LOS compliance for case *i*,*W* *E* *s* *c* *a* *n*_*i*_ indicates a first scan on a weekend for case *i*,*W* *E* *t* *r* *e* *a* *t*_*i*_ indicates a weekend treatment, *χ*_*i*_ is a vector of case-specific characteristics as listed above, and *ε*_*i*_ is an error term. The parameters of interest are the *β*’s, which measure the association between the LOS compliance and weekend scan (*β*_1_) or weekend treatment (*β*_2_), or the combination of both (*β*_3_) relative to weekday scan and treatment. The coefficient *β*_3_ in particular is of main interest because the interaction measures potential gains in LOS compliance if weekend scan and treatment result in more coordinated and efficient services provision. Due to the log-linear functional form of the model, the coefficients can be interpreted as semi-elasticities, i.e., 100*%*∗(*e* *x* *p*(*β*_*j*_)−1) measures the relative change in LOS compliance for weekend scans (*j*=1) or weekend treatments (*j*=2), and 100*%*∗(*e* *x* *p*(*β*_1_+*β*_2_+*β*_3_)−1) measures the relative change in LOS compliance if both scan and treatment are done on a weekend as opposed to a weekday.

For all our analyses, we apply heteroscedasticity-robust (Huber-White) standard errors. We checked for multicollinearity in our explanatory variables using variance inflation factors (VIFs) and obtained a mean VIF in our preferred model (Table 4, column 4) of 2.81 and no VIF larger than 10, and hence we conclude that multicollinearity is not an issue.

## Results

Table 4 presents the main results of our study. The table shows the estimated coefficients of the regression Eq. 2 in four different specifications. The first specification includes indicators for weekend scan and weekend treatment, and the interaction of the two, as well as indicators for weekend admission and discharge. The second specification adds a basic set of controls for the clinical pathway (number of scans, days between scan and admission, between treatment and scan, and between discharge and scan). The third specification also controls for gender, age, and private insurance coverage. Our preferred fourth specification adds indicators for mortality, emergency case, modality, complexity and partition (i.e., internal medicine vs. surgical cases) to the model.

The coefficients of interest for weekend scan and treatment are both very small and statistically insignificant, but the coefficient of the interaction term is statistically significant at the 1% significance level. The latter is relevant for inclusion in the model as highlights Table 5 documenting that its omission would lead to an omitted variable bias. Finally, having both scan and treatment on a weekend is associated with a higher LOS compliance of about

$${((1.06)) + (1.99) - (-19.07))}=22.12\%.^{2} $$

This number is slightly higher in the other three specifications, which indicates that controlling for a basic set of variables that describe the clinical pathway is important to explain LOS compliance (reflected in the adjusted R-squared increasing from 1.7*%* in the first to 35.6*%* in the fourth specification). Regarding the signs of the other coefficients, they are in line with our expectations, but we merely include them as control variables in our model and therefore will not discuss them further.

The last two columns of Table 4 show the results for specification 4 separately by non-emergency and emergency cases. While none of our indicators for weekend scan, weekend treatment or the interaction term is significantly related to LOS compliance in the sample of non-emergency cases, we find a significant association of about the same magnitude as in the overall model for the emergency cases. Hence, the higher LOS compliance observed for weekend scans and weekend treatments is mainly driven by the emergency cases Additional file 1.

**Table 4:** Incremental linear robust models and split data set of emergency and non-emergency cases [37]

	(1) Basic model	(2) Extended model	(3) Model incl.	(4) Model incl.	(5) Emergency	(6) Non-emergency
			patient confounding	all confounders	cases	cases
Constant	0.095^∗∗∗^	0.566^∗∗∗^	0.652^∗∗∗^	0.677^∗∗∗^	0.665^∗∗∗^	0.474^∗∗∗^
	(0.008)	(0.013)	(0.025)	(0.048)	(0.060)	(0.079)
Basic model weekends						
Admission WE	−0.053^∗^	−0.062^∗∗^	−0.060^∗∗^	−0.038^·^	−0.021	−0.066^·^
	(0.024)	(0.021)	(0.021)	(0.020)	(0.025)	(0.035)
Scanning WE	−0.147^∗∗∗^	−0.015	−0.016	−0.011	−0.039	0.056
	(0.029)	(0.025)	(0.025)	(0.025)	(0.031)	(0.044)
Discharge WE	0.160^∗∗∗^	0.033^·^	0.030^·^	0.013	0.035	−0.006
	(0.021)	(0.017)	(0.017)	(0.017)	(0.026)	(0.022)
Treatment WE	−0.104^∗∗^	−0.034	−0.036	−0.020	−0.022	−0.036
	(0.035)	(0.032)	(0.032)	(0.031)	(0.033)	(0.098)
Interaction WE scan and treatment	0.536^∗∗∗^	0.258^∗∗∗^	0.263^∗∗∗^	0.211^∗∗∗^	0.227^∗∗∗^	0.113
	(0.053)	(0.046)	(0.046)	(0.045)	(0.049)	(0.130)
Number of exams		−0.005	−0.006	0.001	0.008^∗^	−0.010
		(0.004)	(0.003)	(0.004)	(0.004)	(0.006)
Diff. scan and admission (in days)		−0.047^∗∗∗^	−0.047^∗∗∗^	−0.048^∗∗∗^	−0.055^∗∗∗^	−0.038^∗∗∗^
		(0.003)	(0.003)	(0.003)	(0.003)	(0.006)
Diff. treatment and scan (in days)		0.004^·^	0.004	0.003	0.005^·^	0.005
		(0.003)	(0.003)	(0.003)	(0.003)	(0.005)
Diff. dismissal and scan (in days)		−0.041^∗∗∗^	−0.040^∗∗∗^	−0.038^∗∗∗^	−0.041^∗∗∗^	−0.035^∗∗∗^
		(0.002)	(0.002)	(0.002)	(0.002)	(0.003)
Patient data						
Female			−0.075^∗∗∗^	−0.080^∗∗∗^	−0.066^∗∗∗^	−0.099^∗∗∗^
			(0.012)	(0.011)	(0.016)	(0.016)
Age			−0.001^∗^	−0.001^∗∗^	−0.002^∗∗∗^	0.000
			(0.000)	(0.000)	(0.000)	(0.001)
Private insurance			−0.011	−0.006	−0.014	0.000
			(0.013)	(0.013)	(0.018)	(0.018)
Clinical data						
Deceased				0.463^∗∗∗^	0.477^∗∗∗^	0.369^∗∗∗^
				(0.043)	(0.049)	(0.089)
Emergency				−0.127^∗∗∗^		
				(0.013)		
Internal medicine case				0.113^∗∗∗^	0.031	0.254^∗∗∗^
				(0.032)	(0.038)	(0.056)
Surgical case				−0.062^∗^	−0.141^∗∗∗^	0.078
				(0.031)	(0.038)	(0.053)
MRI				0.113^∗∗∗^	0.161^∗∗∗^	0.044
				(0.033)	(0.041)	(0.055)
CT				0.081^∗∗^	0.092^∗^	0.073
				(0.030)	(0.037)	(0.051)
X-ray				0.093^∗∗^	0.098^∗∗^	0.093^·^
				(0.030)	(0.038)	(0.050)
Complicated radiology case				−0.106^∗∗∗^	−0.098^∗∗∗^	−0.107^∗∗∗^
				(0.021)	(0.028)	(0.032)
Adj. R^2^	0.017	0.325	0.328	0.356	0.384	0.313
Num. obs.	11371	11371	11371	11371	6234	5137

**Table 5:** Incremental linear robust models highlighting potential omitted variable bias

	(1) Basic model (Omitted variable bias)	(2) Basic model	(3) Extended model	(4) Model incl. patient confounding	(5) Model incl. all confounders
Constant	0.095^∗∗∗^	0.084^∗∗∗^	0.563^∗∗∗^	0.647^∗∗∗^	0.675^∗∗∗^
	(0.008)	(0.008)	(0.011)	(0.024)	(0.049)
Basic model weekends					
Admission WE	−0.053^∗^	−0.063^∗^	−0.066^∗∗^	−0.064^∗∗^	−0.041^·^
	(0.025)	(0.025)	(0.021)	(0.021)	(0.021)
Scanning WE	−0.147^∗∗∗^	0.011	0.061^∗∗^	0.061^∗∗^	0.051^∗^
	(0.032)	(0.028)	(0.023)	(0.023)	(0.023)
Discharge WE	0.160^∗∗∗^	0.167^∗∗∗^	0.035^∗^	0.033^·^	0.015
	(0.020)	(0.021)	(0.017)	(0.017)	(0.017)
Treatment WE	−0.104^∗∗^	0.149^∗∗∗^	0.086^∗∗∗^	0.087^∗∗∗^	0.079^∗∗∗^
	(0.038)	(0.028)	(0.023)	(0.023)	(0.023)
Interaction WE scan and treatment	0.536^∗∗∗^				
	(0.054)				
Number of exams			-0.005	−0.005^·^	0.001
			(0.003)	(0.003)	(0.003)
Diff. scan and admission (in days)			−0.048^∗∗∗^	−0.048^∗∗∗^	−0.049^∗∗∗^
			(0.002)	(0.002)	(0.002)
Diff. treatment and scan (in days)			0.004^∗^	0.004^∗^	0.003^·^
			(0.002)	(0.002)	(0.002)
Diff. dismissal and scan (in days)			−0.041^∗∗∗^	−0.041^∗∗∗^	−0.039^∗∗∗^
			(0.001)	(0.001)	(0.001)
Patient data					
Female				−0.075^∗∗∗^	−0.080^∗∗∗^
				(0.012)	(0.011)
Age				−0.001^∗^	−0.001^∗^
				(0.000)	(0.000)
Private insurance				−0.012	−0.007
				(0.013)	(0.013)
Clinical data					
Deceased					0.464^∗∗∗^
					(0.032)
Emergency					−0.132^∗∗∗^
					(0.013)
Internal medicine case					0.113^∗∗∗^
					(0.034)
Surgical case					−0.065^·^
					(0.033)
MRI					0.115^∗∗∗^
					(0.032)
CT					0.082^∗∗^
					(0.030)
X-ray					0.091^∗∗^
					(0.030)
Complicated radiology case					−0.104^∗∗∗^
					(0.020)
Adj. R^2^	0.017	0.009	0.323	0.326	0.355
Num. obs.	11371	11371	11371	11371	11371

^∗∗∗^ *p*<0.001, ^∗∗^ *p*<0.01, ^∗^ *p*<0.05, ^·^ *p*<0.1 Table indicating heteroskedasticity-consistent robust standard errors and *p*-values with omittted variable bias by excluding the interaction term

One important concern about the above result could be that certain DRGs are more likely emergency cases and also more likely high or low LOS compliance cases. Thus, the DRG classification of the case could confound the relationship between weekend scan and treatment and the LOS compliance. Since we have repeated cases by DRG classification, we can explore the within DRG variation of the data, i.e., we can estimate DRG fixed effects models, which are equivalent to models with indicators for each DRG code in addition to the other controls. Table 6 summarises the results with the specification of the main variables as before. The results indicate that the main effect reported is relatively robust to this alteration of the model. In particular, compared to our preferred specification 4 in Table 4 we find a sligthly higher LOS compliance effect of 24.27*%* for weekend scans and treatments. The result is still mainly driven by the emergency cases, i.e., there seem to be similar mechanisms at play also within the DRG codes, or stated differently, when the confounding influences of the different case-mix are adjusted for in the regression model.

**Table 6:** Incremental DRG fixed effects models with and without emergency cases

	(1) Basic model	(2) Extended model	(3) Model incl. patient confounding	(4) Model incl. all confounders	(5) Emergency cases	(6) Non-emergency cases
Basic model weekends						
Scanning WE	−0.181^∗∗∗^	−0.079^∗∗∗^	−0.080^∗∗∗^	−0.071^∗∗∗^	−0.061^∗^	−0.079^∗^
	(0.030)	(0.021)	(0.021)	(0.021)	(0.027)	(0.038)
Treatment WE	−0.077^∗^	−0.008	−0.009	0.005	0.011	0.015
	(0.036)	(0.025)	(0.025)	(0.025)	(0.029)	(0.058)
Discharge WE	0.175^∗∗∗^	0.084^∗∗∗^	0.079^∗∗∗^	0.062^∗∗∗^	0.074^∗∗∗^	0.043^∗^
	(0.020)	(0.014)	(0.014)	(0.014)	(0.020)	(0.018)
Admission WE	−0.044^·^	−0.031^·^	−0.032^·^	−0.016	−0.006	−0.005
	(0.024)	(0.017)	(0.017)	(0.017)	(0.021)	(0.029)
Interaction WE scan and treatment	0.462^∗∗∗^	0.200^∗∗∗^	0.203^∗∗∗^	0.191^∗∗∗^	0.160^∗∗∗^	0.107
	(0.051)	(0.036)	(0.036)	(0.036)	(0.042)	(0.087)
Number of exams		−0.046^∗∗∗^	−0.045^∗∗∗^	−0.037^∗∗∗^	−0.034^∗∗∗^	−0.041^∗∗∗^
		(0.003)	(0.003)	(0.003)	(0.004)	(0.005)
Diff. scan and admission (in days)		−0.063^∗∗∗^	−0.063^∗∗∗^	−0.062^∗∗∗^	−0.068^∗∗∗^	−0.055^∗∗∗^
		(0.002)	(0.002)	(0.002)	(0.003)	(0.003)
Diff. treatment and scan (in days)		0.007^∗∗∗^	0.007^∗∗∗^	0.008^∗∗∗^	0.014^∗∗∗^	0.001
		(0.001)	(0.001)	(0.001)	(0.002)	(0.002)
Diff. dismissal and scan (in days)		−0.051^∗∗∗^	−0.051^∗∗∗^	−0.050^∗∗∗^	−0.056^∗∗∗^	−0.041^∗∗∗^
		(0.001)	(0.001)	(0.001)	(0.001)	(0.001)
Patient data						
Female			−0.038^∗∗∗^	−0.041^∗∗∗^	−0.044^∗∗^	−0.039^∗∗^
			(0.010)	(0.010)	(0.014)	(0.013)
Age			−0.003^∗∗∗^	−0.003^∗∗∗^	−0.003^∗∗∗^	−0.003^∗∗∗^
			(0.000)	(0.000)	(0.000)	(0.000)
Private insurance			0.009	0.006	0.008	0.023
			(0.011)	(0.010)	(0.015)	(0.014)
Clinical data						
Deceased				0.355^∗∗∗^	0.351^∗∗∗^	0.150^∗∗^
				(0.029)	(0.035)	(0.057)
Emergency				−0.120^∗∗∗^		
				(0.012)		
Internal medicine case						
Surgical case						
MRI				−0.007	−0.010	0.009
				(0.028)	(0.037)	(0.045)
CT				0.011	0.004	0.001
				(0.025)	(0.033)	(0.041)
X-ray				0.069^∗∗^	0.052	0.099^∗^
				(0.025)	(0.033)	(0.042)
Complicated radiology case				−0.143^∗∗∗^	−0.148^∗∗∗^	−0.123^∗∗∗^
				(0.017)	(0.023)	(0.025)
R^2^ (proj model)	0.019	0.501	0.506	0.520	0.539	0.468
Num. obs.	11371	11371	11371	11371	6234	5137
R^2^ (full model)	0.244	0.616	0.619	0.630	0.667	0.666

^∗∗∗^ *p*<0.001, ^∗∗^ *p*<0.01, ^∗^ *p*<0.05, ^·^ *p*<0.1 Table indicating clustered standard errors including emergency and non-emergency cases We report R^2^ instead of Adj. R^2^ because of the enhanced collinearity through the DRG fixed effects model. Leading to a negative Adj. R^2^ that would have to be interpreted as 0

## Discussion

This study describes the relationship between scan and treatment day and LOS compliance in a Swiss university hospital. Our results suggest that having both scan and treatment on a weekend is positively related with the LOS compliance. Our main interpretation of this finding is that standardised operating procedures are in place on weekends to scan and treat emergency patients, which seem to be more efficient than corresponding procedures on weekdays. In this section, we briefly discuss potential reasons why the day of the week should matter for the LOS compliance and possible implications of our findings.

Earlier studies have been able to show similar patterns, or at least no differences, in the quality of service levels comparing weekends and weekdays, especially for ICU units, which are oftentimes closely related to emergency cases [30, 31]. Innovative work organizations and staff models are two potential explanations for maintaining higher service levels for patients [32]. Arriga et al. [32] also shows that standardised operating procedures, and particularly checklists, positively influence clinical results, especially for weekend patients. Lawrence et al. [33] have shown how an efficient resource use on weekends could lead to optimized results, e.g., not prolonging hospital stays unnecessarily. However, the evidence on the potential channels explaining higher service levels on weekends versus weekdays is still scarce, and further research is needed to assess the exact reasons for higher quality care in specific environments.

Overall, this area of research appears to be promising in gaining a better understanding of the potential inefficiencies in the provision of inpatient services (see for example [34] for an overview). It seems to be of particular relevance in areas where emergency procedures apply. Mohammed [35] provides evidence for weekend admissions (split into elective and emergency cases) being associated with an increased risk of death, with slightly stronger associations found for elective cases. The results of this type of work are relevant for other health care institutions as well, where a better coordination of services could improve clinical and economic outcomes. Currently, this area is not strongly researched and further investigations for example for ambulatory care institutions, large physician practices or long-term care institutions could provide valuable insights.

Our results are also relevant from a public health perspective. Using a small back-of-the-envelope calculation, we illustrate that by improving the LOS compliance at the above rate, one can for example reduce the number of required inpatient beds within a health care region. Table 7 indicates that the infrastructure savings could be substantial if the found weekend efficiencies would be applied to the overall health care system’s number of hospital beds. Assuming a generally accepted cost for a newly built hospital bed in Switzerland of about 1 million CHF this would mean approximately 2^′^312 million CHF^4^ that could be saved in the overall health care system in the years to come. Compared to the total number of available inpatient beds, we can observe a reduction of nearly one third (including excess capacities) that could be achieved applying weekend procedures to all weekdays.

**Table 7:** LOS compliance: effect estimate of process adoption on inpatient beds

	Patient days (PD)	ALOS	Beds
Total patients CH	1^′^123^′^995	6.2	
OFSP^3^ 2012	6’944’354		23’852
Required beds by calculation(ALOS*Nbr. of Patients/365)			19’026
General bed utilization			79.8*%*
Required emergency beds only (54.8% of the study cases)			10^′^426
Efficiency gains (only on emergency beds and based on total weekend effect)		−1.37	2’312
Required beds incl. Efficiency		4.83	16’714
Bed utilization (incl. efficiency gains only on emergency beds)			70.1*%*

Total Patient Days = ALOS * PD The number of beds is extracted from the OFSP statistics

Since an inefficient use of hospital beds is likely a driver of growing health care costs, our results offer input for the development of more coordinated health care service plans. Together with the progress in medical technologies this could have the potential to reduce health care spending for inpatient services significantly. Of course, the applicability of weekend procedures to all weekdays still needs to be assessed. Our study also has several limitations. First, the scope of the analysis is limited to a single university hospital because the data linkage of DRG cases with information from the RIS is not available on the level of the medical statistics of all hospitals, as for example provided by the Swiss Federal Office of Statistics, which would encompass administrative records of all DRG cases in Switzerland. Moreover, we confined the analysis to a particular aspect of the clinical pathway, namely radiological intervention and treatment day. We did not analyse the full pathway because of the complexity of the different combinations of scan days, modalities and treatments. We also have limited information about the quality of the clinical pathway, except for the LOS compliance, and hence we could not support our argument of more efficient and standardized procedures with our own data. We also lack information about staff schedules and skill sets within the weekend or regular weekday team setups. Different working styles or even methods on weekends could be influencing factors, which are part of our explanation of the higher LOS compliance with coordinated radiological intervention and treatment on weekends. Unfortunately, our data does not allow us to disentangle the different explanations, which would require additional information from the human resource systems and detailed background of the hospital staff. Finally, and related to the previous point, radiological interventions and treatments were not randomised, i.e., we cannot make causal claims about our results. Rather, we interpret our analysis as providing a meaningful association between LOS compliance and scan/treatment day, which is robust to a number of potential confounders, including DRG-specific heterogeneity.

## Conclusion

Our study is the first that looks at the interaction between radiological intervention and treatment day and how this is related to LOS compliance in a DRG hospital in Switzerland. We find a positive and significant association between weekend scan and treatment and the LOS compliance. This result has immediate implications for hospital management since it is indicative of more efficient clinical pathways on weekends, or put differently, potential efficiency gains that could be achieved by applying the same processes on all days of the week. Although we cannot pinpoint the sources of the differences in LOS compliance between weekday and weekend scans and treatments, standardised operating procedures in place on weekends, and their application compliance are a likely explanation. Of course, further research is needed to investigate the clinical pathways in greater detail. Our results also have implications for the health care system in general. Managing scarce hospital resources, radiologic technology and human resources, in the best way seems to be beneficial in terms of both hospital performance and clinical outcomes, as reflected in a higher LOS compliance. This can lead to substantial cost savings in the health care infrastructure, as shown by our rough cost calculations in Table 7 above. Learning at best from existing processes and using their full potential could be a response to the increased cost pressure on public decision makers in planning health services provision, regional cooperation between hospitals just one example of such a strategy.

## Endnotes

^1^ For our purpose we explicitely simplify the then existing systems acknowledging other specifications that are not relevant for our discussion.

^2^ The formula for the definition of the effect size per dummy is derived from [36]

100[*e*(−*c* ^∗^−1/2∗*v* ^∗^(*c* ^∗^))−1]

where *c* ^∗^ is the estimated value and *v* ^∗^corresponds to the square of the standard error for *c* ^∗^). As our dummy moves from weekend to working day, i.e., from 1 to 0, the factor (−1) is required.

^3^ Office fédéral de la Santé Publique = Federal Office of Public Health.

^4^ Using the efficiency gains only in emergency beds in Table 7 and mutiplied with the approximate price of a newly built Swiss hospital bed.

## Additional file

Additional file 1 LOS Compliance with Scan and Treatment on Weekends split in Emergency and Non-Emergency. (PNG 441 kb)
