# Supplementary figures and images for: Is the timing of radiological intervention and treatment day associated with economic outcomes in DRG-financed health care systems: a case study
Source: BMC Health Serv Res. 2017 Feb 28;17:168. doi: 10.1186/s12913-017-2055-0 (PMC5329950; doi:10.1186/s12913-017-2055-0)

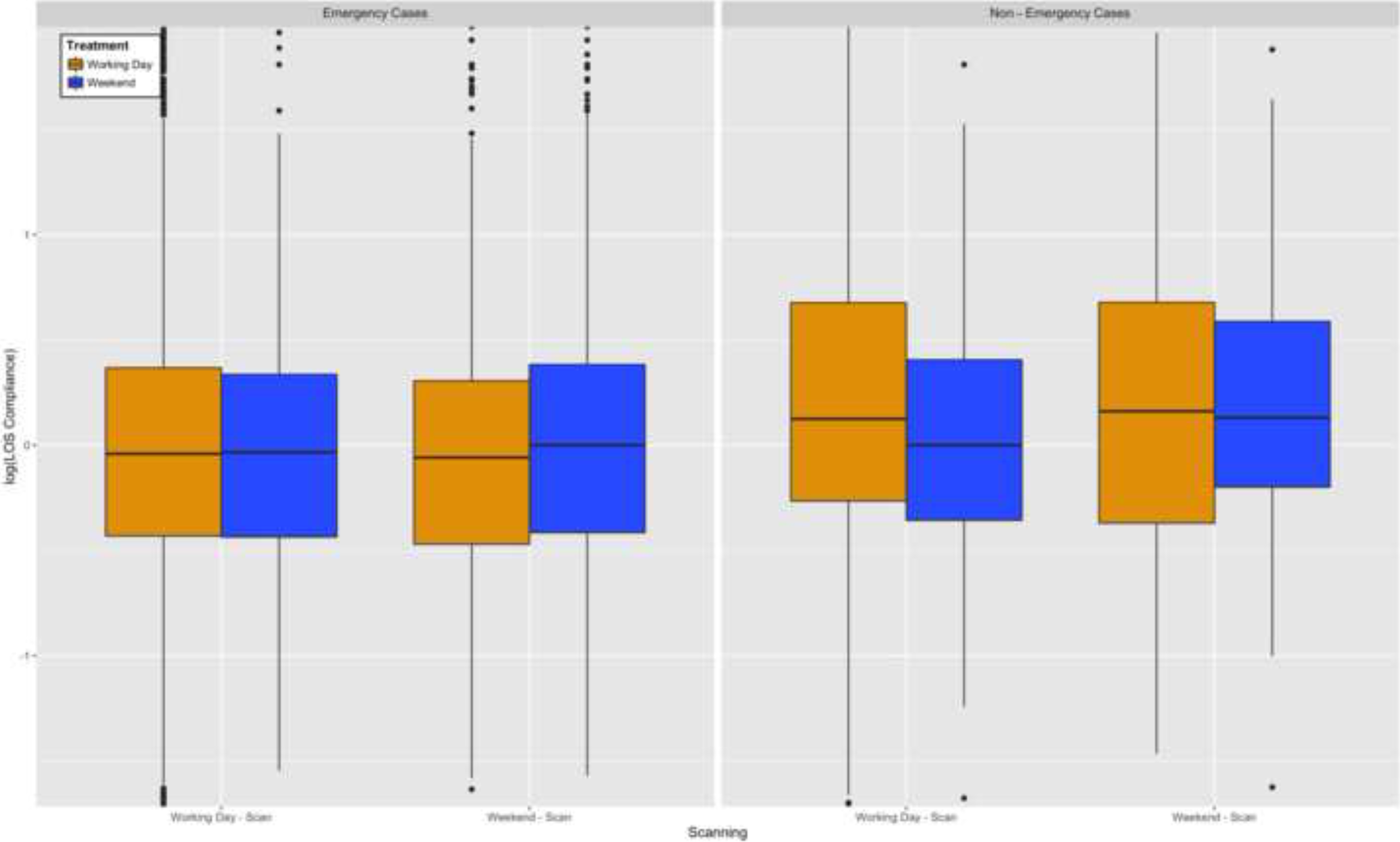

Supplement: Additional file 1 — LOS Compliance with Scan and Treatment on Weekends split in Emergency and Non-Emergency. (PNG 441 kb) [file 12913_2017_2055_MOESM1_ESM.png]
